# “A shared enemy to confront“: the experience and needs of the partners of kidney transplant patients - a qualitative study

**DOI:** 10.3389/ti.2026.16491

**Published:** 2026-07-29

**Authors:** Katerina Kyriakouli, Anna Yakinthou, Smaragdi Marinaki, Christina Papachristou

**Affiliations:** 1 Department of Psychology, Aristotle University of Thessaloniki, Thessaloniki, Greece; 2 Department of Medicine, Ethniko Kai Kapodistriako Panepistemio Athenon, Athens, Greece

**Keywords:** chronic illness, kidney transplantation, needs, partners’ experiences, qualitative study, relationship

## Abstract

Kidney disease and transplantation affect not only the patients but also their partners, requiring couples and families to adapt to and to cope with complex and ongoing psychosocial and practical demands. Despite playing a crucial role in caregiving and transplant success, partners’ experiences and needs often remain overlooked. This study aimed to explore the lived experiences and needs of partners of kidney transplant recipients in the specific socio-cultural context of the study. Twelve in-depth interviews with partners of kidney transplant patients were conducted and analyzed using thematic analysis. The following themes were identified: 1) The secondary presence: Partners in the background, 2) From Illness to a New Normal, 3) The Need for Support and Clearer Communication from Healthcare Professionals, 4) Redefining the “We” and 5) Transplantation as a Life-Changing Lesson. Findings indicate that partners overtake emotionally complex roles supporting their spouses, yet their difficulties and needs often remain invisible. While going through significant challenges during critical phases of kidney disease, they experience transplantation relationally, through dyadic coping. The shared couple’s experience necessitates a focus on partner’s voices and needs as well as a holistic approach aiming at managing and adapting couples to the transplantation process.

## Graphical Abstract

**Figure:**
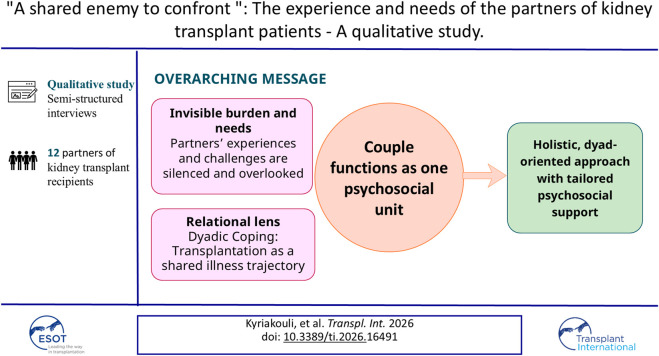

## Introduction

Chronic kidney disease (CKD) is a progressive condition characterized by persistent abnormalities in kidney structure or function, associated with accelerated cardiovascular disease, severe infections and premature death [1]. The global prevalence of CKD is increasing mainly due to the pandemic of obesity and type 2 diabetes, affecting more than 10% of the global population [2]. Haemodialysis (HD) is the most common form of kidney replacement therapy. Although technology and patient access to HD have advanced significantly, impaired quality of life, morbidity and mortality rates remain high [3]. Kidney transplantation is regarded as the optimal treatment offering enhanced survival and improved quality of life in comparison with HD [4].

CKD and kidney transplantation affect not only the people with kidney failure but also their partners. Partners face significant psychological distress, such as anxiety and depression due to their spouse’s illness and transplantation [5, 6]. Partners of people in dialysis experience considerable physical and psychological burden due to constant responsibility overload. They often struggle with overwhelming demands associated with caregiving, balancing complex practical and emotional-stressing roles, and uncertainty about the patient’s health [7–10].

The impact of kidney transplantation on partners, has been studied primarily quantitatively focusing on post-transplant psychological status and dyadic coping. Dyadic coping is positively associated with mental and physical quality of life of both patient and caregiver [11], emotional functioning, wellbeing and relationship quality and satisfaction [12, 13]. Following kidney transplantation, partners report reduced caregiver burden, anxiety, depression [14–16], and improvements in overall quality of life, sleep quality, social life, sexual relationships, and stress [17]. However, Rodrigue et al. found that most partners still experience high strain after kidney transplantation, likely due to ongoing post-transplant care or comorbidities [18].

Few qualitative studies investigate the experiences of partners of kidney transplant recipients. Hann et al. studied the experiences of partners of simultaneous pancreas-kidney recipients, who reported persistent worry about their spouses health, enhanced responsibilities and neglect of their own health and wellbeing with an immediate effect on their health, wellbeing, relationships and work [19]. Following transplantation, partners felt relief and return to normality, but still experienced stress related to recovery and potential complications. Researchers emphasise that more in-depth qualitative research regarding the impact of kidney transplantation on partners is needed understand the refined complexities of their situation and to address gaps in understanding how they are affected [7, 8, 20]. Furthermore, in-depth data that highlight the nuances among different cultural backgrounds are of importance. The study seeks to provide a deeper understanding of the experience of the—largely overlooked in research—partners of deceased or living kidney transplant recipients, in the specific cultural environment of Greece, with the ultimate aim to highlight the partners’ voices and formulate adequate support interventions.

The research questions were:

1. How did the partners of people who have undergone kidney transplantation experience their spouse’s illness?
2. What is the perceived impact of illness and transplantation on the relationship?
3. What are the partner’s needs in managing the illness and the transplant process?

## Materials and methods

### Design

A qualitative research design was chosen to obtain detailed, in-depth information. Twelve semi-structured interviews were conducted with partners of kidney transplant recipients and analysed using thematic analysis.

### Sample and recruitment

Purposive sampling was applied in order to intentionally include individuals based on predetermined inclusion criteria allowing for depth of insight. Data collection ended when data saturation was achieved. Participants were recruited via announcements on social media forums for kidney transplant patients and websites of the Panhellenic Association of Kidney Patients and the Association of Transplant Recipients of Magnesia, Greece. Additional recruitment took place through the Kidney Transplant Unit of Laiko General Hospital, in collaboration with an independent nephrologist. Interested individuals could contact the interviewers to receive information on the study and clarify questions before arranging an interview. We decided to focus on partners who are not living kidney donors of the patients. Being a donor partner raises specific type of complexities and dynamics that would need in our opinion to be studied in a separate sample.

Inclusion criteria were 1) age 25 years or older, 2) relationship with the recipient before and during the transplantation period, 3) ability to provide informed consent and 4) sufficient use of the Greek language. The minimum age criterion of 25 years was set to ensure sufficient emotional maturity to discuss sensitive topics and increase the likelihood of established, longer-term relationships. Given the typical age profile of this population, this criterion was not expected to substantially restrict eligibility.

### Setting

Interviews were conducted between December 2022 and May 2023. Five interviews were conducted online and seven took place in participants’ homes.

### Materials

A semi-structured interview guide was developed following literature review and team discussions, consisting of 30 open-ended and non-directive questions. The interview guide addressed: 1) the history and experience of kidney disease and the transplantation process, 2) the couple’s relationship, 3) the partner’s role, 4) personal needs, and 5) future expectations.

### Procedure

Interviews were conducted by two research team and lasted an average of 55 min. The interviews were audio-recorded and transcribed verbatim by the researchers.

### Data analysis

Data were analyzed using thematic analysis due to its flexibility and capacity to identify patterns of meaning [21]. A flexible application of Brown and Clarke’s stages was conducted. First, researchers familiarized themselves with the data through repeated listening, transcript reading and recording of notes. Then, initial codes were generated, grouped, and refined to ensure internal consistency. Sixty-five initial categories were merged into 14, from which five overarching themes emerged. An inductive approach was used, with most codes developed at a latent level and some at a semantic level. Representative excerpts were selected to illustrate themes, ensuring coherence with the research questions and existing literature.

Quality criteria proposed by Braun and Clarke were followed through all the stages, regarding transcription, coding and analysis. The reporting of this qualitative study followed Consolidated Criteria for Reporting Qualitative Research (COREQ) [22], as detailed information, recommended by the COREQ checklist, regarding the research team and reflexivity, study design, data analysis and findings reporting is provided. Reflective support from the project supervisors, experienced in the transplantation field, and the use of a reflective journal throughout the process, enhanced analytical depth and research quality [21].

### Ethics

Ethical approval was obtained by the Ethics Committee of the Aristotle University of Thessaloniki and the Scientific Council of the Laiko General Hospital of Athens. The research complied fully with the guidelines of the European Union General Data Protection Regulation (GDPR, effective 28 May 2018). All participants provided informed consent and full confidentiality was maintained.

## Results

### Participant characteristics

The final sample consisted of N = 12 participants (9 females, 3 males; mean age = 55 years, 6 = employed, 3 = unemployed, 3 = retired). All participants were of Greek ethnicity. Participants’ characteristics are presented at Table 1. All recipients underwent dialysis before transplantation and received kidneys from either living (LKDT) or deceased donors (DCD). None of the partners were donors. Participants’ characteristics are presented at Table 1.

**TABLE 1:** Participant characteristics.

Partner					Kidney transplant recipient		
Participant	Sex	Age	Professional status	Family status	n transplantation	Donor type	Duration in dialysis
Maria	F	40	Employed	Married, with children	1	D	1.5 years
Anna	F	46	Employed	Married, with children	2	D	13 years
Markos	M	74	Retired	Wife deceased, children and grandchildren	1	L	1 year
Dimitra	F	65	Retired	Husband deceased, with children and grandchildren	2	D	7 months [1] 10 years [2]
Ioanna	F	55	Unemployed	Married	1	D	7 years
Vasilis	M	58	Employed	Married, with children	1	D	12 years
Christina	F	73	Retired	Married with children and grandchildren	1	D	5 years
Melina	F	41	Unemployed	Married, with children	2	L [1] D [2]	8 months [1] 13 years [2]
Katerina	F	56	Employed	Married, with children	1	L	9 years
Giorgos	M	59	Employed	Married, with children	1	D	6 years
Stella	F	55	Unemployed	Married, with children	1	L	3 years
Despina	F	37	Employed	Married, with children	1	L	6 months

Participant names are pseudonyms to protect confidentiality. No. of transplantations, Donor type, and Duration of dialysis refers to the characteristics of the person with kidney failure.

**FIGURE 1:**
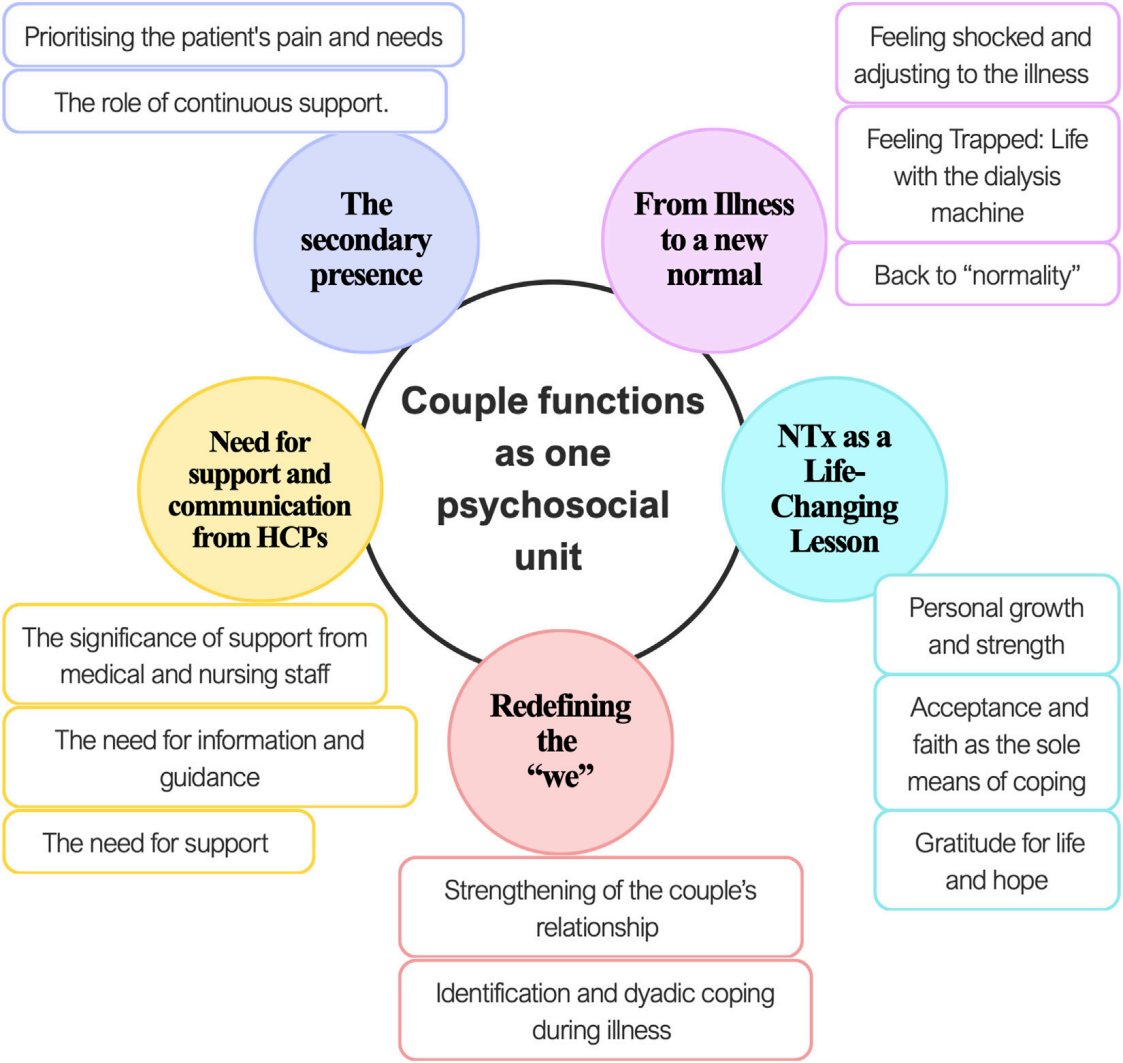
Schematic overview of the main findings. *Note*. Schematic overview of the five main themes (circles) and their associated sub-themes (boxes), illustrating the couple as a single psychosocial unit throughout the kidney transplantation journey. NTx = Kidney transplantation; HCPs = healthcare professionals.

Five broader themes were identified: 1) The secondary presence: Partners in the background, 2) From Illness to a New Normal, 3) The Need for Support and Clearer Communication from Healthcare Professionals, 4) Redefining the “We” and 5) Transplantation as a Life-Changing Lesson. Figure 1 provides a schematic overview of the main findings.

#### The secondary presence: partners in the background

##### Prioritising the patient’s pain and needs

Participants consistently minimized their own psychological difficulties, challenges and needs, and considered the patients’ physical suffering as more legitimate and significant than their own. Feelings of guilt were reported for experiencing distress because it is *“the patient (who) goes through it all”.* The recipient’s emotions are prioritized over the partners emotions as the recipients’ physical pain seems to weigh more: “*Six years in dialysis means her hand was pierced 1,872 times. What can I possibly say? Talk about my own feelings? Her feelings are what matters the most.”* (Giorgos). The majority of participants downplayed their contribution to caregiving, while some admired their partners’ strength thinking of the people with CKD partially as a “*hero*”. Partners adapted their behaviour and emotional expression according to their partner’s physical and psychological condition and compromised in order not to burden or destabilize them. For many participants that meant suppressing negative emotions, avoiding also the expression of joy, as it felt inappropriate compared to their partner’s suffering. *“This is the right thing to do, you should not start showing your feelings. You should not express your sleepiness, your hunger, your pain, your desire to cry, or even your joy. Especially if the other person is in pain”*(Maria). Through transplantation, partners reported not reflecting on their own psychological state as they focused on their loved-onesbody and survival, emphasizing the need for strength and endurance describing a state of survival. “*I was in survival mode. In other circumstances I might have wanted to cry, sit alone and think about it, even break down. But you go into a mode of ‘no, now we survive, we focus on the body”* (Ioanna). *These things have to be done, and we will endure everything”.*

##### “You have to be present”: the role of continuous support

The majority of participants described that their duty as partners is to be constantly present and to provide emotional and practical support. They reported assuming more responsibilities and new roles, including management of daily routines, household tasks, organizing visits to the hospital and medical examinations, ensuring medication adherence and compliance with medical guidelines. Female partners in particular reported greater burden and difficulty in balancing their overlapping roles as mothers, workers and caregivers. “*I reached a point where I had my job, my kids, dealing with his illness, and at the same time managing everything in the house. I wasn’t able to sleep because I was working nights. I basically had to carry it all on my shoulder*” (Dimitra) Many participants felt responsible for their partners’ health and expressed regret or even guilt for not being more assertive in their role. *“You need to put some pressure on him about these health issues. I wish I had pushed my husband harder to go to the doctors, to take things more seriously. Maybe we would not have reached the point we did. Sometimes I blame myself, thinking I should have been more insistent”* (Christina).

#### From illness to a new normal

##### Feeling shocked and adjusting to the illness

The experience of kidney disease and dialysis initially evoked feelings of stress, anxiety and fear in most participants. Participants expressed concerns regarding the risks involved in dialysis treatment and about their partner’s condition after dialysis. Several felt unprepared and unaware of the seriousness of the illness and its difficulties experiencing the diagnosis of end stage kidney disease but also the commencing hemodialysis as “traumatic” and shocking: *“When I first learned about this diagnosis, I was in such shock that for 3 days I could not recognize familiar faces”* (Katerina).

##### Feeling trapped: life with the dialysis machine

Life on dialysis was experienced as extremely restrictive, as the couple were constrained due to the lengthy and frequent visits which limited their daily activities and travel. Many participants mentioned their partner’s physical weakness after treatment as additionally restricting and unfair, dialysis as “*a life and death machine*” and transplantation appealed as an “*escape*”. *“Υou cannot go on vacation or even go out, because 1 day he was on dialysis and the next day he was too tired. We did not travel for about seven and a half years. I watch my neighbour and my sister going on trips, while I cannot do the same”* (Vasilis).

##### Back to “normality”

Transplantation was experienced by many partners with great relief, as liberation and with a sense of freedom to regain normality, ability to travel, and go out. “*Our lives have changed. Now, we are free. Daily life is simpler and easier. We live just like any other family would. There’s no difference.“*(Stella, 55, F). *Despite the relief,* eight partners expressed anxiety and stress regarding organ rejection, health complications, side-effects of immunosuppressive medication and infections. Fear of infections led several pairs to limited social interactions and loss of important relationships. *“After transplantation, we started to worry about infections, that one of us might catch a virus. Ηe should not be in close contact with people. We even lost contact with our grandchildren*!” (Christina). After successful transplantation, when their partner’s health was stabilized, some participants reported that they “broke down”. Specifically, two participants reported symptoms of exhaustion and burnout, while two individuals experienced symptoms of depression, attributing that to the accumulation of suppressed emotions.“*At the beginning I was very strong, I was not afraid, I had a lot of faith, I did not have any psychosomatic symptoms. After the first year of the transplantation, though, I started having very intense symptoms of fear and depression”* (Katerina).

#### The need for support and transparent/precise communication from healthcare professionals

##### The significance of support from the medical and nursing staff

Many participants stressed the importance of support, reassurance, and maintaining a ‘human’ attitude on the part of doctors. Some participants recollected memories with healthcare professionals that functioned as a source of strength, courage and hope throughout the course of their illness. Feeling safe and held by the team appeared as extremely crucial for coping: *“I really admired the way the transplant team managed everything. I never imagined they could do all that. Personally, the fact that these people made me feel so safe was enough. I did not need anything else”* (Stella).

##### The need for information and guidance

Nine participants highlighted the need for information and guidance from healthcare professionals on managing the disease pre and post transplantation. They referred to reduced communication and interaction with healthcare professionals, reporting a lack of information regarding the course of treatment, hemodialysis, post-transplant monitoring, and the partner’s health status and complications. For the participant who lacked guidance, support and safety this feeling of being left “*alone after transplantation*” was described as the hardest part of the process. “*Not knowing how everything is going, not having the support of a doctor, someone who can guide on a daily basis and explain why things are the way they are*” (Dimitra) This need for security and guidance was expressed by some participants also in regards to a wish for greater involvement of the healthcare team in decision-making.

##### The need for support

While many participants described supportive healthcare professionals, some participants mentioned impersonal and distant encounters with healthcare professionals, emphasising the lack of psychological support and empathy and the bitter impact on them.*“From the doctors, personally, I did not get anything, no help at all. I do not even think they ever spoke to me, to be honest [laughs]”* (Anna). Many participants expressed a need for psychological support, highlighting the absolute necessity to improve the welfare system, the state’s support structures and the in-hospital care system. *“Ideally, a psychologist should come to talk with both the patient and the family, because this is a huge change for everyone. We were all emotionally affected, both as a family and the patient. This did not happen”* (Stella).

#### Redefining the “we”

##### Strengthening of the couple’s relationship

Most participants described being satisfied with their relationship before the illness and that the transplantation process had strengthened the couple’s relationship. Partners reported greater intimacy, emotional contact and gratitude for their relationship.*“I think that it helped and brought us even closer, because these kinds of situations can make you come closer to the other person, they cannot drive you apart. And even without a transplant, couples today have a thousand reasons to grow apart. Α transplant will never be the reason”* (Dimitra). Seven participants reported that there was no change in their sexual activity, which remained stable or even improved according to three participants. Astonishingly, several participants reported receiving social reactions that ranged from encouragement to leave their ill spouse to expressions of fear that the partner might abandon them or be unfaithful. In all cases, participants rejected these ideas and emphasized their love and commitment.

##### Identification and dyadic coping during illness

Many participants perceived their partners’ illness as something they were going through themselves also, empathizing with their experience. *“It felt like I was going through it myself. Didn’t matter if it was him or me, it was the same”* (Anna). Notably, the majority of participants used the first-person plural when describing medical procedures. Furthermore, partners described that they managed illness and transplant process as a single unit, in the sense of dyadic coping. They emphasized the importance of mutual support and collaboration between the couple. Kidney disease was experienced as a “shared enemy”: *“It’s something we’re going through together—like we’re fighting a shared enemy. It feels like we’re facing it side by side. So we’ll beat it, will not we? That way, we’ve got a better chance”* (Despina).

#### Transplantation as a life-changing lesson

##### Personal growth and strength

After going through the experience of illness and transplantation, partners report a feeling of personal growth. The transplant process and their caregiving role gifted them with a sense of strength and confidence for future challenges, while some partners reported feeling wiser, more patient and less selfish and describe this journey as “a life lesson”, reporting: “*I came out stronger and wiser because I learned to be much more patient. It was something that really challenged us. After every struggle, you come out stronger, you do not come out defeated. I clearly feel stronger. It was a life lesson”* (Despina).

##### Αcceptance and faith as the sole means of coping

Acceptance of the situation was regarded as the sole means of coping by most of the participants. Since illness, dialysis treatment, and the finding of a donor were out of their control, participants reported reconciling themselves to the situation, using humor, staying calm and optimistic about the future. “*During the waiting period, I came to accept it. I kept saying, “They’ll find an organ when the time comes.” It was not an obsession. When the moment comes, it will come. We did not lose ourselves over it”* (Maria). Four people also mentioned the value of faith in God in managing illness and trust in something beyond them *“(we) left it to God, whatever he wants, so be it”*.

##### Gratitude for life and hope

Lastly, the transplantation process evoked feelings of gratitude, appreciation for life and recognition of the value of health, reflecting on how fragile, but at the same time precious it is. Partners described to have developed a positive and optimistic attitude towards life and reported a renewed hope. *“It helped shape me, made me see things differently, to realize that it's silly to get caught up in small stuff, to let my mind dwell on it and get upset, and that there are more important things than that. And of course, I’ve learned to truly appreciate the value of health, on the highest level”* (Dimitra).

## Discussion

This qualitative study explored the experience and needs of partners of people who have undergone kidney transplantation, an often overlooked group despite their central role incoping, adherence and overall transplant process. The overarching message emerging from the findings is that the transplant recipient and partner function as one psychosocial unit. The illness trajectory is experienced dyadically, indicating that partners are actively involved, suffer in parallel-sometimes silently- and carry the emotional weight on behalf of their partner. Despite this, partners have little space to express their distress, receive support, or be acknowledged within healthcare services, making them the “invisible heroes” of the journey.

This dyadic perspective resonates with the concept of the “we-desease” [23] and the framing of the person with kidney failure and their partner as a psychosocial dyad [24]. Across all themes, partners described sustained involvement in all phases of their spouse’s chronic illness pre-, during and post-transplantation, highlighting that kidney disease is not an individual challenge but a shared experience. Thus, transplantation is framed through a dyadic lens, involving emotional support, empathy, joint problem-solving [25, 26].

Although kidney disease and transplantation were experienced as a shared journey, partners’ own experiences remained largely invisible, as reflected in the theme *The secondary presence: Partners in the background.* Aligning with previous research, partners often suppress their emotions and significantly downplay their contribution to caregiving [27–32]. This was also reflected with Kim and Lee’s notion of “the fading of caregiver’s own life” [33].

This pattern may reflect the moral and social framing of caregiving as an obligation [34, 35], together with healthcare systems that promote patient-centered focus [36]. Caregivers in a recent study reported feeling excluded and undervalued by healthcare teams, despite their contribution, indicating graft-centered care [37]. The study’s results emphasize how transplant teams should attend to partners’ neglected experiences, encourage self-care and prevent a mental and physical collapse. Within this context, partners’ unmet needs remained largely explicitly voiced, highlighting the need to uncover’ implicit needs during critical phases of the transplant trajectory, such as the dialysis and the waiting list period.

Different challenges marked different critical phases, as it emerged from the second theme, *From Illness to a New Normal*. Dialysis was seen by participants as the most burdensome stage, characterized by anxiety and uncertainty, consistent with previous research [31, 38–40]. For partners, dialysis represented a period of vulnerability and significant restrictions, with transplantation representing both relief and an escape from constraints.

Transplantation was initially experienced as liberation bringing relief and restored normality in daily functioning aligning with earlier findings [15–17]. However, many partners also experienced stress about organ rejection and complications and some experienced emotional strain or decline in wellbeing after the transplantation. This was also reflected in Rodrigue’s et al. study, showing that partners experience considerable caregiving strain both before and after transplantation [18], whereas a recent study reported family members’ anxiety and uncertainty about transplant failure and sudden death of their loved-one [41].

These mixed findings suggest that the post-transplant period may represent a second critical phase for partners. While the immediate caregiving demands decrease, their suppressed emotions and needs surface. This may reflect shifts in their role—from caregiver to spouse [42] —which can bring both relief and emotional collapse as the need to remain strong subsides [32, 43]. These findings underscore the importance of investigating partners’ outcomes over different phases of the process in order to develop tailored interventions targeted at stage-specific challenges.

Adding a unique perspective, findings highlighted that these challenges were experienced relationally through processes of dyadic coping. The fourth theme, *Redefining the We*, showed that illness often strengthened relationships, fostering communication, emotional closeness, and mutual support. In similar findings, couples report high relationship satisfaction after transplantation [44], while the shared experience of the illness has been reported to lead to new dimensions of intimacy, a stronger bond, and a sense of fulfillment [34, 40, 45]. Participants viewed illness as a shared challenge through dyadic coping, involving emotional support, empathy, joint problem-solving [25, 26].

However, the broader literature presents a more nuanced picture, with several studies reporting relationship strain and sexual relationship dissatisfaction [17, 46, 47]. The lack of negative reports may reflect volunteer bias, as those with a positive relationship were more willing to participate [48] or may have avoided discussing sensitive topics such as sexuality.

Assessing couples’ relationships before transplantation may help design interventions that strengthen dyadic coping and adaptation. Since dyadic coping predicts partner’s wellbeing and adjustment [44], it likely contributed to relationship satisfaction and adaptation observed. Future research should focus on both partners’ perspective using mixed methods to better understand shared adaptation and inform couple focused interventions.Given partners’ central role in the transplantation process as part of a psychosocial dyad, support and information needs emerged. Consistent with previous studies, partners often lacked sufficient knowledge about the illness, dialysis, caregiving, and post-transplant care [37, 43, 49–51]. Unmet information needs can negatively affect partners’ wellbeing and caregiving ability [52, 53], highlighting the importance of tailored guidance. Healthcare professionals should provide individualized information and prepare partners for caregiving, considering each family’s needs and capacities [54]. Educational materials and programs on kidney disease and its treatments could further address these gaps. Future research should assess information needs, and evaluate educational interventions.

Despite significant burden, transplantation was experienced *as a Life-Changing Lesson,* reflecting personal growth, resilience, and gratitude. Participants emphasized acceptance, optimism, and faith, developing inner strength and appreciation for life, consistent with post-traumatic growth and resilience [55, 56]. With studies highlighting positive aspects of caregiving [7, 57, 58], these insights promote that meaning-making and resilience could enhance psychosocial support for partners.

This study has several limitations. As a qualitative study, findings are based on subjective interpretation and are not generalizable. The small, predominately female and heterogeneous sample limited exploration of subgroup differences (e.g., rural vs. urban). The sample appeared highly motivated, and relationally stable, often reporting good pre-existing relationships and successful transplant outcomes. Partners with fewer coping resources or less supportive relationships may experience significantly greater distress and remain underrepresented.Experiences of individuals in newer relationships or after separation may also differ. Finally, the limited discussion of challenges and unmet needs, may have restricted the full understanding of partners’ experiences.

## Conclusion

This study demonstrates that kidney transplantation is not solely an individual medical event but a shared, relational experience that affects both recipients and their partners. Partners take over a central yet often underrecognized role, carrying significant emotional, practical and relational responsibilities while consistently prioritizing the recipient’s needs over their own. These findings highlight the need for a holistic, dyad-oriented, approach that includes partners in care and provides tailored psychosocial support across all stages of the transplant journey. Implementing couple-based interventions may enhance adaptation and wellbeing for both partners and people with CKD.

## Data Availability

The raw data supporting the conclusions of this article will be made available by the authors, without undue reservation.
